# A Rare Case of Autoimmune Pulmonary Alveolar Proteinosis Developing During the Course of Eosinophilic Granulomatosis With Polyangiitis

**DOI:** 10.1002/rcr2.70219

**Published:** 2025-05-20

**Authors:** Yoichi Dotake, Kentaro Tanaka, Shiro Fujisaki, Kenichi Shimobaba, Hirotoshi Kuroiwa, Midori Satomura, Hiromi Matsuyama, Koichi Takagi, Hideo Mitsuyama, Hiromasa Inoue

**Affiliations:** ^1^ Department of Pulmonary Medicine Graduate School of Medical and Dental Sciences, Kagoshima University Kagoshima Japan

**Keywords:** asthma, eosinophilic granulomatosis with polyangiitis, pulmonary alveolar proteinosis

## Abstract

Eosinophilic granulomatosis with polyangiitis (EGPA) is an anti‐neutrophil cytoplasmic antibody (ANCA)‐associated vasculitis characterised by asthma, eosinophilia, and systemic inflammation, often involving the lungs. We present the case of a 47‐year‐old woman with EGPA who developed progressive ground‐glass opacities and a crazy‐paving pattern on chest computed tomography (CT). Bronchoalveolar lavage revealed milky fluid, and transbronchial lung biopsy showed periodic acid‐Schiff (PAS)‐positive eosinophilic granular material. Elevated anti‐granulocyte‐macrophage colony‐stimulating factor (GM–CSF) antibodies confirmed a diagnosis of autoimmune pulmonary alveolar proteinosis (aPAP). Corticosteroid tapering initially led to EGPA relapse, which was successfully controlled with mepolizumab, enabling further steroid reduction. Following this, the radiological findings of aPAP showed gradual improvement. In rare cases, it is known that autoimmune diseases such as vasculitis can be complicated by aPAP. This case highlights the importance of individualised immunomodulatory treatment and close imaging follow‐up in patients with overlapping autoimmune conditions.

## Graphical abstract

Here, we report a rare case in which autoimmune pulmonary alveolar proteinosis (aPAP) developed during the clinical course of eosinophilic granulomatosis with polyangiitis (EGPA). To our knowledge, this is the first reported case of its kind. We present the clinical features and discuss potential mechanisms based on the literature.

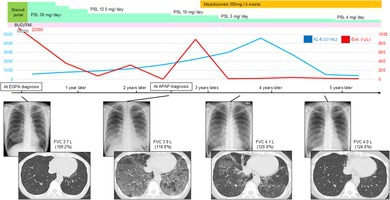

## 1 Introduction

Eosinophilic granulomatosis with polyangiitis (EGPA) is a systemic eosinophilic vasculitis classified as an anti‐neutrophil cytoplasmic antibody (ANCA)‐associated vasculitis. It is typically preceded by allergic conditions such as asthma or allergic rhinitis and involves eosinophilic infiltration of multiple organs, mononeuritis multiplex, purpura, and systemic symptoms due to vasculitis. Pulmonary manifestations include eosinophilic pneumonia, pleuritis, and alveolar haemorrhage. Here, we report a rare case in which autoimmune pulmonary alveolar proteinosis (aPAP) developed during the clinical course of EGPA. To our knowledge, this is the first reported case of its kind. We present the clinical features and discuss potential mechanisms based on the literature.

## 2 Case Report

A 47‐year‐old woman with a history of childhood‐onset bronchial asthma developed exertional dyspnoea and was hospitalised for further evaluation. She had been receiving regular inhaled budesonide/formoterol since she was 34 years old, with stable control. At the age of 44, she developed bilateral leg numbness and purpura, accompanied by marked peripheral eosinophilia (approximately 20,000/μL). Electrophysiological testing was consistent with mononeuritis multiplex, and a skin biopsy revealed perivascular eosinophilic infiltration. Although ANCA was negative, the clinical findings and pathology met the diagnostic criteria for EGPA. She was treated with steroid pulse therapy, followed by oral prednisolone (PSL) 50 mg/day, with rapid reduction in eosinophil count. PSL was tapered over time.

**FIGURE 1:**
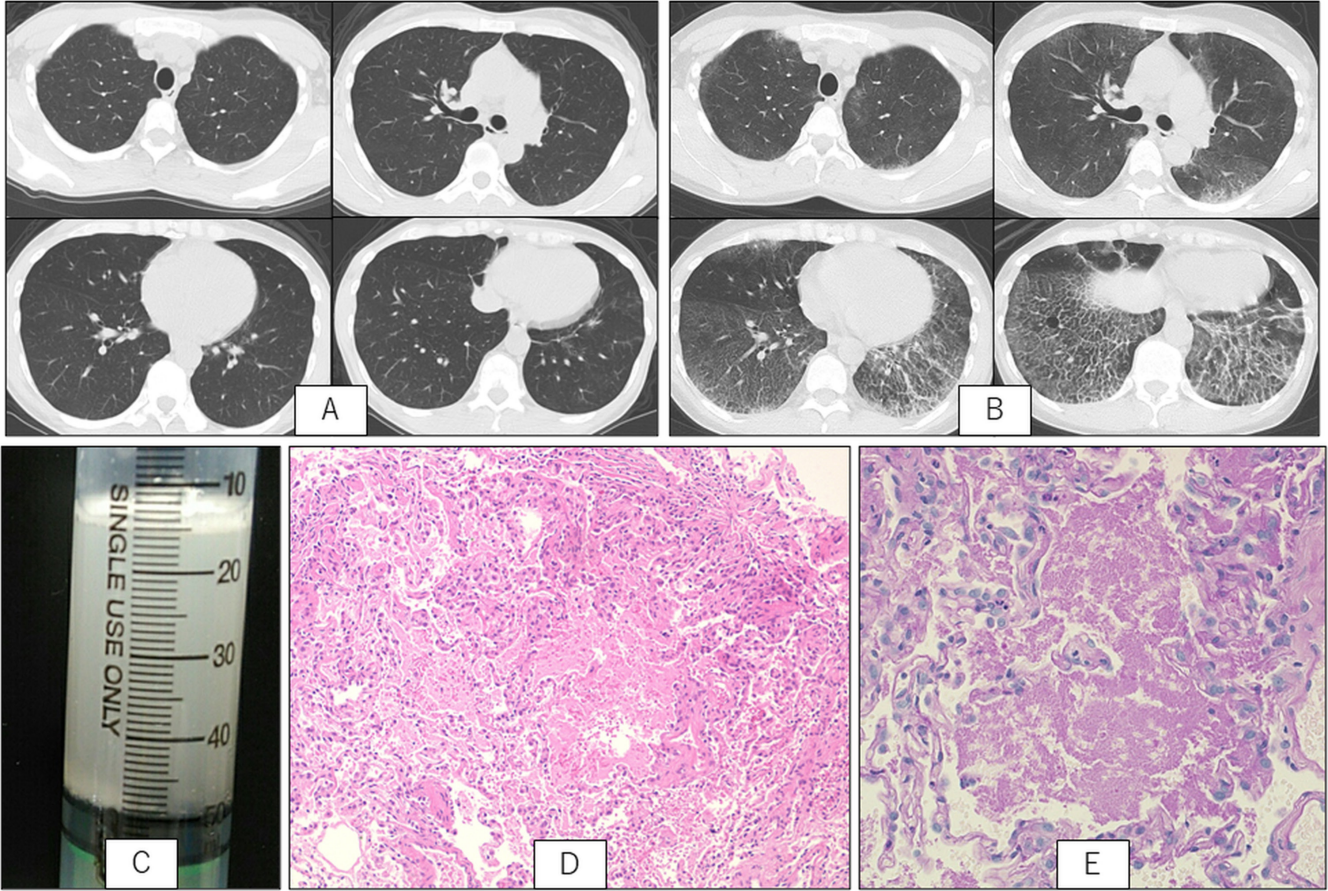
(A) Chest computed tomography (CT) at the time of eosinophilic granulomatosis with polyangiitis (EGPA) diagnosis showing faint bilateral ground‐glass opacities (GGOs). (B) Chest CT at the time of autoimmune pulmonary alveolar proteinosis (aPAP) diagnosis showing scattered GGOs in both lungs, with interlobular septal thickening mainly in the lower lobes, resulting in a crazy‐paving pattern. (C) Bronchoalveolar lavage fluid (BALF) appeared milky. (D) Transbronchial lung biopsy (TBLB) specimen showing fine eosinophilic granular deposits within the alveolar spaces (haematoxylin and eosin stain, ×100). (E) The deposits were positive on periodic acid–Schiff (PAS) staining (PAS stain, ×200).

At the time of EGPA diagnosis, chest computed tomography (CT) showed faint bilateral ground‐glass opacities (GGOs), which were initially attributed to EGPA‐related lung involvement (Figure 1A). Bronchoscopy was not performed at that time. Over time, the GGOs and reticular opacities, mainly in the lower and central lung fields, worsened. Interlobular septal thickening also appeared predominantly in the lower lobes, forming a crazy‐paving pattern (Figure 1B).

Laboratory testing showed no elevation in eosinophils or total IgE. KL‐6 was mildly elevated at 761 U/mL. Myeloperoxidase (MPO)–ANCA and proteinase 3 (PR3)–ANCA were negative. Arterial blood gas analysis on room air revealed a pH of 7.41, PaCO_2_ of 40.8 Torr, and PaO_2_ of 71.3 Torr, with an HCO_3_‐level of 25.2 mmol/L. The calculated alveolar–arterial oxygen gradient (A–aDO_2_) was mildly elevated at 27.4 Torr. Pulmonary function testing showed normal spirometric values (VC 113% predicted, FVC 121% predicted, FEV_1_ 123% predicted, FEV_1_/FVC ratio 83.6%), but a mildly reduced diffusing capacity for carbon monoxide (DLCO 63.2% predicted). Bronchoscopy was then performed to investigate possible infection, pulmonary alveolar proteinosis (PAP), or eosinophilic pneumonia. Bronchoalveolar lavage (BAL) from the right B^5^ bronchus yielded a milky, turbid fluid (Figure 1C). The BAL fluid showed 56.0% lymphocytes and 23.0% eosinophils. Transbronchial lung biopsy (TBLB) from the right B^2^, B^4^, and B^8^ showed eosinophilic granular material in the alveolar spaces, positive for periodic acid‐Schiff (PAS) staining (Figure 1D,E). The amount of anti–granulocyte‐macrophage colony‐stimulating factor (GM–CSF) antibodies in the serum was elevated at 46.5 U/mL, leading to a diagnosis of aPAP.

**FIGURE 2:**
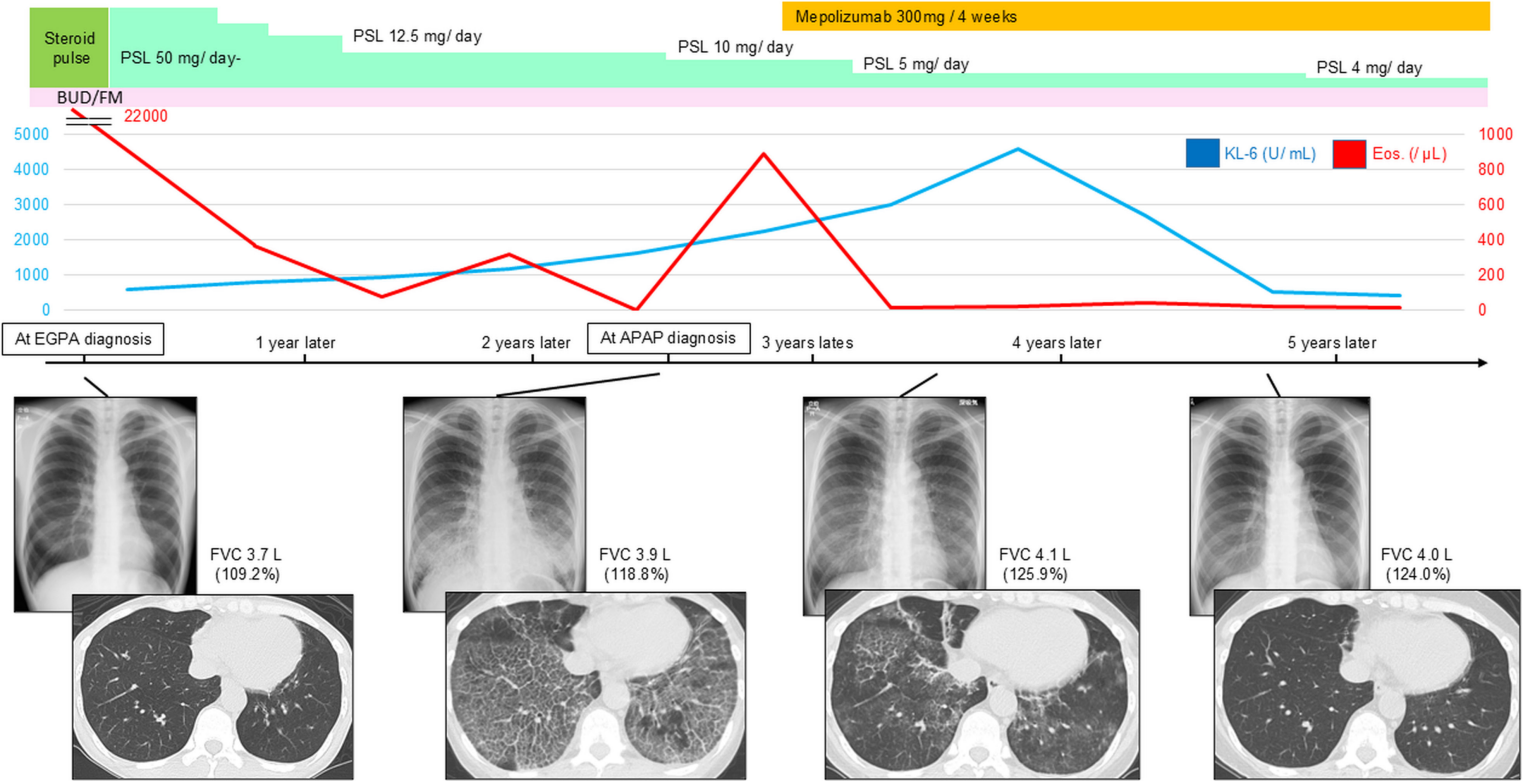
Clinical course of the patient. After the diagnosis of autoimmune pulmonary alveolar proteinosis (aPAP), tapering of corticosteroids was attempted. However, this led to an increased eosinophil count, indicating the worsening of eosinophilic granulomatosis with polyangiitis (EGPA) disease activity. Mepolizumab was subsequently initiated, resulting in a rapid decrease in eosinophil count. With continued steroid tapering, chest CT findings gradually showed marked improvement.

Given that the patient's pulmonary symptoms were relatively mild and her oxygenation was only mildly impaired, we opted for close monitoring and adjustment of anti‐inflammatory treatment rather than initiating GM‐CSF inhalation therapy or total lung lavage. At that time, the patient was on PSL 12.5 mg/day for EGPA, which was tapered to 10 mg/day after the aPAP diagnosis. However, this led to an increased eosinophil count. Mepolizumab (300 mg every 4 weeks) was initiated to control EGPA activity and facilitate further steroid tapering. The eosinophil count declined promptly. Follow‐up imaging showed gradual improvement in pulmonary opacities. Three years later, the patient currently remains stable on PSL 4 mg/day and mepolizumab, with preserved respiratory function and no systemic symptoms (Figure 2).

## 3 Discussion

EGPA is an ANCA‐associated vasculitis that primarily affects small vessels. Pulmonary involvement occurs in approximately 50%–70% of patients with EGPA and may present with transient infiltrates, pleural effusions, or non‐cavitating nodules [1]. In the present case, the initial GGOs were suspected to represent pulmonary manifestations of EGPA. However, the progression to a crazy‐paving pattern raised suspicion of PAP, which was confirmed via bronchoscopy and histology.

The crazy‐paving pattern is non‐specific and can be seen in various diseases such as infections, neoplasms, and acute respiratory distress syndrome (ARDS) other than PAP. It has also been reported that vasculitis and eosinophilic pneumonia could present with similar patterns [2]; therefore, examination by bronchoscopy is essential for making a diagnosis. In this patient, the combination of milky BAL fluid, PAS‐positive material on TBLB, and elevated anti‐GM–CSF antibodies supported a diagnosis of aPAP.

PAP is caused by impaired surfactant clearance due to dysfunctional alveolar macrophages. aPAP, which accounts for 90% of PAP cases [3], is mediated by anti‐GM–CSF antibodies. In contrast, secondary PAP may be associated with hematologic disorders, environmental exposures, infections, autoimmune diseases, or certain drugs. Although secondary PAP was initially suspected in our patient, antibody positivity confirmed the autoimmune type.

In a national Japanese survey, autoimmune diseases were present in 1.4% of patients with aPAP [3]. Only one case of microscopic polyangiitis with aPAP has been reported in which aPAP preceded vasculitis [4].

GM–CSF levels are elevated in several autoimmune diseases and may serve as a biomarker of disease activity in EGPA. In a reported case of aPAP with systemic sclerosis and sarcoidosis, increased GM–CSF production due to autoimmune activity was proposed to induce anti‐GM–CSF antibody production [5]. Similarly, in our patient, active EGPA may have triggered antibody formation, leading to aPAP.

PAP patients are vulnerable to opportunistic infections because of macrophage and neutrophil dysfunction. Therefore, the use of corticosteroids for autoimmune diseases can exacerbate this immunosuppression and can be considered a poor prognostic factor in PAP. In our case, given the immunosuppressive risks and the potential for spontaneous improvement in mild cases, we opted to adjust anti‐inflammatory treatment with close monitoring. Mepolizumab, a targeted drug for IL‐5, effectively suppressed EGPA disease activity, which allowed PSL to be reduced and may also help to improve aPAP.

To our knowledge, there is currently no evidence that mepolizumab directly influences the production of anti–GM‐CSF antibodies. However, by effectively controlling eosinophilic inflammation and facilitating corticosteroid tapering, mepolizumab may help stabilise the immune environment in patients with aPAP. Although the precise mechanism remains unclear, this case suggests that mepolizumab could serve as a beneficial adjunctive therapy in patients with coexisting EGPA and aPAP.

This rare case highlights that careful monitoring and appropriate intervention are crucial when managing complex autoimmune overlaps. It also suggests that treating the underlying autoimmune process—potentially responsible for excess GM–CSF production—plays an important role in controlling aPAP in such a context.

## Author Contributions

Yoichi Dotake and Kentaro Tanaka wrote the manuscript. All authors critically revised the manuscript for important intellectual content and approved the final version of the manuscript.

## Ethics Statement

The authors declare that written informed consent was obtained for the publication of this manuscript and accompanying images using the form provided by the Journal.

## Conflicts of Interest

Hiromasa Inoue reports grants from Boehringer Ingelheim, GlaxoSmithKline, and Omron, as well as personal fees from AstraZeneca, Boehringer Ingelheim, GlaxoSmithKline, Kyorin, and Sanofi, outside of the submitted work. The other authors declare no conflicts of interest.

## Data Availability

The data that support the findings of this study are available from the corresponding author upon reasonable request.
